# The Good Manager: Development and Validation of the Managerial Interpersonal Skills Scale

**DOI:** 10.3389/fpsyg.2021.631390

**Published:** 2021-03-29

**Authors:** Gerard Beenen, Shaun Pichler, Beth Livingston, Ron Riggio

**Affiliations:** ^1^Department of Management, College of Business and Economics, California State University, Fullerton, CA, United States; ^2^Department of Management and Entrepreneurship, Tippie College of Business, The University of Iowa, Iowa City, IA, United States; ^3^Department of Psychology, Claremont McKenna College, Claremont, CA, United States

**Keywords:** managerial abilities, scale validation, scale development, multilevel modeling, managerial interpersonal skills, supervisor support, motivation, conflict management

## Abstract

It is no secret that employees leave their organizations because of bad managers- but what about the good ones? How can researchers and organizations differentiate individuals in terms of the interpersonal skills needed to perform well in the managerial role? Although these are fundamentally important questions to organizational psychologists, there exists no conceptual model, definition, or measure of interpersonal skills specific to the managerial role. We address these questions and research gaps by developing a conceptual model and validating a concomitant measure of managerial interpersonal skills (MIPS) through a research program that included four studies across three phases: First, through a review of the literature and structured interviews with practicing managers; next, three quantitative studies in which we develop, refine and validate our MIPS scale; and finally, in a fourth validation study with matched supervisor-employee data from a large healthcare organization. Results suggest that MIPS are best represented by a three-dimensional model comprised of supporting, motivating and managing conflict all indicating a higher-order latent MIPS factor. Results also indicate the MIPS Scale predicts job attitudes and performance among both employees and managers above and beyond personality traits and leader-member exchange, as well as constructs closely related to MIPS, such as social support and conflict management style.

## Introduction

In his popular guide to *Building a civil workplace, and surving one that isn’t*, Robert Sutton describes the differences between good and bad managers:

Bosses shape how people spend their days and whether they experience joy or despair, perform well or badly, or are healthy or sick. Unfortunately, there are hoards of mediocre and downright rotten bosses out there, and big gaps between the best and the worst (Sutton, 2007, p.34).

Managing people is inherently demanding and stressful. Managers must coordinate and oversee the work of others in the context of constrained resources, changing demands and expectations, and perhaps most significantly, challenging interpersonal exchanges. Recent studies have suggested that managers experience generally high levels of stress due to job demands (Cavanaugh et al., 2000), which can lead to emotional exhaustion and burnout. This raises the issue of how to manage employees most effectively from an interpersonal perspective. Since managers spend most of their work hours in interpersonal exchanges (e.g., Rubin and Dierdorff, 2009), managerial interpersonal skills (MIPS) are integral to being a “good boss.”

Indeed, scholars have suggested that interpersonal skills, or “skills employed when persons interact with one another” (Klein et al., 2006, p. 81), are essential to organizational and managerial effectiveness, and individual career success and employability (Tyson, 2020). For instance, employee interpersonal skills are related to attitudes and performance at the individual and team levels, and ultimately to firm productivity, and have predicted long-term job performance in healthcare settings (Lievens and Sackett, 2012). Interpersonal skills are also among the most in-demand skills for success in organizations and for managerial effectiveness (Graduate Management Admissions Council, 2005; Beenen et al., 2018; Rios et al., 2020). Google recently concluded that skills such as coaching others and communicating effectively were more important than science, technology, engineering and math (STEM) skills for so-called Googlers’ performance (Glazer, 2018). This should not be surprising as organizations rely on extensive social interaction, are increasingly reliant on effective service, yet often lack managers with the interpersonal skills necessary for success (e.g., Klein et al., 2006). The importance of interpersonal skills in work settings is amplified by our increasing reliance on digital communication channels—especially among younger generation workers—which can impair effective work relationships (Kick et al., 2015). Despite the critical role of managerial interpersonal skills, no published study has produced a comprehensive definition, conceptual model, and a validated scale to measure *managerial interpersonal skills* (MIPS). The purpose of this study, therefore, is to provide such a definition and conceptual model, and to develop and validate the MIPS Scale.

We report on a three phase systematic research program that includes four studies: In phase one, we did an extensive review of the literature to determine which dimensions a MIPS scale might include. In phase two, we generated items based on our literature review as well as interviews with practicing managers and executives. We then tested and refined a preliminary MIPS measure (Study 1). This was followed by further testing of a refined measure and set of items, as well as tests of convergent and criterion-related validity (Study 2). We then integrated input from thought leaders to further refine, finalize and validate the scale items^1^ (Study 3). Phase three focused on our final validation study with multi-level manager-subordinate matched data from a large healthcare organization (Study 4). The goal of this phase was to build on and address the limitations of the prior three studies and further validate our MIPS Scale. Our multiphasic, multi-sample approach is consistent with scale development and validation studies published in top managerial psychology journals (e.g., Stewart et al., 2009).

## Theoretical Background and the MIPS Construct

Job demands-resources (JD-R) theory (Bakker and Demerouti, 2014) synthesizes principles from the work motivation and job stress literatures and provides a useful framework for why managerial interpersonal skills may be related to important outcomes for managers and employees, namely job attitudes and performance. JD-R theory organizes psychosocial work characteristics into two categories: job demands and resources (Demerouti et al., 2001). Job demands are work characteristics that require sustained psychological or physiological effort and thus psychological or physiological costs. Job resources are physical, psychological, social or organizational work characteristics that reduce job demands and costs; help workers achieve work goals; and stimulate learning, growth and development (Demerouti et al., 2001; Schaufeli and Bakker, 2004; Bakker and Demerouti, 2014). Job resources stimulate employee motivation to the extent they satisfy basic psychological needs such as belongingness or competence and thus are positively related to job attitudes (Schaufeli and Bakker, 2004).

Research on JD-R theory has shown that job resources, such as social support, can reduce stress and strain experienced on the job due to various job demands (Demerouti et al., 2001). Indeed, social support from colleagues (Bakker and Demerouti, 2014) and supervisors has played a prominent role in the job-demands resources literature (Kossek et al., 2011). General social support is the extent to which one provides emotional support (care for one’s well-being) and instrumental support (resources needed to complete work-related tasks) (Kossek et al., 2011). Supervisors in particular are instrumental in providing the esteem, feedback and support that are so important to employee attitudes and performance (Demerouti et al., 2001; Bakker and Demerouti, 2007; Beenen et al., 2017). We expect MIPS also may be a potential overlooked resource in the JD-R literature that may be broader than supervisory support and predictive of job attitudes and performance for both employees and managers.

Increasing the availability of workers’ job and personal resources is especially important in a modern economy with increased job demands due to work intensification^2^ (Macky and Boxall, 2008). Managers also experience high levels of job demands and stress (Cavanaugh et al., 2000), due, for instance, to emotional exhaustion. Interpersonal skills may provide managers themselves with additional resources they can rely on to improve their own performance and quality of life. The question we address with this research program is “what are the dimensions of interpersonal skills that matter most in the managerial role?” Our overarching proposition is that MIPS function as a key job resource for both employees and managers and, as such, should predict their job attitudes and performance (Bakker and Demerouti, 2017, 2018). The support and feedback that employees receive from interpersonally skilled managers should support their basic psychological needs, thereby improving job attitudes and performance (Schaufeli and Bakker, 2004). Interpersonally skilled managers should experience more positive job attitudes and performance, because interpersonal skills are an essential resource for the effective management of people (Bakker and Demerouti, 2017).

Our overarching proposition is consistent with research on job characteristics which has shown that social skills are one of four key dimensions of job performance, as well as research that has shown some skills that are interpersonal in nature (e.g., political skills), are a key resource that help employees reduce stress by gaining control over work events (e.g., Perrewé et al., 2000, 2005). Our propositions are also in line with the concept of bandwidth fidelity from the psychometric theory literature, which calibrates the breadth of antecedents to their corresponding outcomes (e.g., Hogan and Holland, 2003). In other words, narrower antecedents should predict narrower outcomes, and broader antecedents should predict broader outcomes. Because we conceptualize MIPS as broader in bandwidth than related constructs such as social support, MIPS should have a stronger relationship to broad outcomes such as job attitudes and performance^3^ (Kossek et al., 2011; French et al., 2018).

We began our research program with a review of the literature on workplace interpersonal skills, and managerial interpersonal skills in particular. Our goal was to assess the status of the MIPS construct in the existing literature, and explore which factors or dimensions it might include. Later, we return to JD-R theory to help us assess the criterion-related validity of our MIPS measure based on our conceptual model.

## Phase I: Literature Review

Social scientists have been interested in interpersonal skills for decades. For instance, Thorndike introduced the concept of “social intelligence,” as a trait consisting of the “… ability to act wisely in social relations” (Thorndike, 1920). More recent approaches propose interpersonal skills are correlated with and influenced by traits, but are nevertheless learned, trainable and expressed through behaviors (Riggio, 1986; Klein et al., 2006). Research on interpersonal skills has been characterized as “piecemeal, poorly conceptualized, and surprisingly lacking in rationale” (Spitzberg and Cupach, 2011, p.503) due to the broad array of skills, microskills and frameworks classified as interpersonal in nature. In the most comprehensive review of workplace interpersonal skills to date, Klein et al. (2006) examined 58 frameworks encompassing over 400 general interpersonal skills, and proposed organizing them into two higher-order dimensions with 12 subdimensions: communication (active listening, oral, written, assertive, non-verbal) and relationship development (trust, intercultural sensitivity, service orientation, self-presentation, social influence, conflict resolution and negotiation).

Klein et al.’s (2006) focus, however, was general workplace interpersonal skills, not managerial interpersonal skills. For example, it is not clear whether service orientation applies to a managerial role in the same way as it might to a service employee. It also is not clear whether written communication, though important, is distinctly interpersonal. Furthermore, two dimensions may not be specific enough to be useful for practical purposes such as selection or training and development. The over 400 micro-skills identified by Klein et al. (2006) and others are too numerous for an actionable model of managerial interpersonal skills that is useful to managers, applied psychologists, and HR practitioners. Since the focus of our research is managerial interpersonal skills, we turn to the literature on the managerial role.

### The Managerial Role and Managerial Interpersonal Skills

Mintzberg (1980) proposed managers occupy three key interrelated roles: informational, decisional, and interpersonal. The interpersonal role involves maintaining relationships with internal and external stakeholders, and motiving employees. Mintzberg’s model also has been framed as three skills sets critical for managerial effectiveness: technical (informational), conceptual (decisional) and interpersonal (e.g., Dierdorff et al., 2009). Of these, research suggests that interpersonal skills are the most critical (e.g., Abraham et al., 2001), especially at the executive level (Kaplan and Sorensen, 2017), and are highly sought after by employers of MBA graduates (Bedwell et al., 2014; Beenen et al., 2018). Managers spend most of their time interacting and communicating with peers, subordinates and their managers (Rubin and Dierdorff, 2009). Therefore, interpersonal skills are important for managers of all tenure ranges and levels, whereas technical skills are relatively more important for newer and less experienced managers, and conceptual skills are relatively more important for more senior-level managers (Mumford et al., 2007). Interpersonal skills are comparatively more predictive of success in the managerial role regardless of tenure and level (e.g., Scullen et al., 2003).

Despite this affirmation of the importance of interpersonal skills for managers, researchers have yet to develop a conceptual model, definition or validated measure of MIPS. Measures of social skills (Riggio, 1986), communication skills (Riggio et al., 2003), and political skills (Ferris et al., 2005) are certainly relevant for managers, though not specific to the managerial role. Thus, these constructs likely do not represent a broader bandwidth construct domain of MIPS, which presents construct validity issues in terms of using these measures as proxies for MIPS. As such, these measures are thus potentially not as predictive of managerial success or subordinate outcomes posited by JD-R theory such as job attitudes and performance (Hogan and Holland, 2003).

Alternatively, models of leader competencies provide a starting point from which to better understand the construct domain of MIPS. The leadership strataplex model (Mumford et al., 2007), for instance, provides a model and measure of four broad leadership skills: cognitive, interpersonal, business, and strategic. Mumford et al. (2007) found that leaders in higher level positions require higher levels of each skill, and that some skills, e.g., strategic skills, were more likely to emerge among leaders in higher positions. This is a useful model that suggests interpersonal skills are essential to effective leadership. That said, the purpose of the Mumford et al. (2007) study was not to develop a model or measure of MIPS. Their data were based on O^∗^NET^4^ assessments, and their measure of interpersonal skills included only four items, each measuring a different dimension of interpersonal skills (e.g., social perceptiveness, persuasion). In our view, this may be appropriate in the context of a broader set of leadership skills. In assessing MIPS, one item per dimension may not provide a valid assessment of a broad bandwidth MIPS construct.

Rubin and Dierdorff (2009) also developed a model of leader competencies based on O^∗^NET data^5^ focused on two categories: managing human capital (coaching and developing others, resolving conflicts and negotiation, developing/building teams) and managing the task environment (communicating outside the organization, establishing/maintaining interpersonal relationships, selling/influencing). Similar to the leadership strataplex model, the model of managerial behavioral competencies developed by Rubin and Dierdorff (2009) included other components as well, such as managing strategy and innovation. In a different paper, Dierdorff et al. (2009) found that the social context of managerial work (i.e., work role context) is more strongly related to interpersonal role requirements than conceptual and technical/administrative role requirements.

While these studies have helped advance the literature on leader competencies, there are some limitations with these models for conceptualizing MIPS specifically. For instance, in the Rubin and Dierdorff (2009) model, communication is limited to “outside the organization” and some interpersonal skills are under a higher-order dimension not exclusive to the interpersonal role (managing the task environment). Moreover, neither Mumford et al. (2007) nor Rubin and Dierdorff (2009) aimed to develop a model or measure of MIPS; their focus was on broader leadership competencies. The current state of MIPS research is reflective of broader scholarship on interpersonal skills that has been portrayed as “challenging and sometimes frustrating” (Spitzberg and Cupach, 2011, p.488) due to the lack of an agreed upon conceptual model and measure (Bigelow, 2015; Riggio, 2020).

Nonetheless, the existing literature on workplace interpersonal skills, the managerial role, and leadership competencies provides a useful starting point when it comes to developing a model of MIPS. Specifically, some common themes emerge that may provide a foundational understanding of what MIPS may include, namely interpersonal communication, relationship development, persuasion and influence, and conflict management. We leveraged this literature and these themes in our item development, as we elaborate below. With that said, existing models of leadership competencies are just that—models of the broad competencies leaders need to be successful in their role. These are not models of MIPS, nor do they provide a validated measure of the MIPS construct. Our intention was to develop a model that is specific to the managerial role, reflects specific skills managers can develop, and is accordingly useful to researchers and organizations for applied purposes, captures the construct domain, and is not deficient.

To that end, our next goal was to compare and augment the extant themes noted above (communication, relationship development, persuasion and influence, conflict management), by providing managers a *tabula rasa* opportunity (i.e., using unaided recall) to identify interpersonal skills that they think matter most in their roles. This ensures our MIPS model and concomitant measure will be informed by the existing literature and is also based on practicing managers’ experience. The extant literature may not be completely consistent with actual practicing managers’ experience, and we wanted to ensure that our model and measure were not deficient. Without a validated model and measure of MIPS, scholars are likely talking about different constructs across studies that refer to MIPS. Furthermore, our goal was to determine if MIPS are a broader bandwidth construct that provides job resources for managers consistent with the JD-R model, as opposed to other narrower bandwidth related skills, such as supervisory support.

## Phase 2: Developing and Testing a Preliminary Measure of Managerial Interpersonal Skills (MIPS)

Given that our literature review revealed a lack of a model and measure of MIPS, the next part of our research program involved interviewing practicing managers and executives as key informants to complement our literature review. This allowed us to develop items for the MIPS Scale based on (1) the existing literature, (2) our knowledge of the construct, and (3) the experiences of professional managers. We conducted in-depth semi-structured interviews with an Institutional Review Board (IRB) approved minimal risk protocol and participant consent for a sample of 27 practicing managers and executives whom we contacted directly or through professional referral. Participants included middle to senior executive level managers from a variety of sectors working in organizations with an average size of *M* = 17,528 employees. Participants had an average of 21.3 years of work experience, 7.4 direct reports in their current positions, and were 23 percent female. Our goal was to understand the potential dimensional structure of interpersonal skills from a managerial perspective. We relied on unaided recall to reduce biased responses by simply asking interviewees to “please list up to seven interpersonal skills that you, based on your experience, consider critical to being an effective manager.” After the list was generated, we followed up with probes by asking each interviewee to elaborate on the nature of each listed skill, and to provide a positive or negative example of each skill.

Interviews were recorded, transcribed, and content analyzed to identify consistent higher order themes across interviewees. This resulted in five higher-order themes: *managing-self*, *communicating*, *supporting*, *motivating*, and *managing conflict*. At least two researchers coded sub-themes and specific skills under each of these five higher-order themes. Based on our review of the literature, our knowledge of the MIPS construct, as well as our interviews with practicing managers, we generated an initial pool of 100 items with about 20 items per sub-theme. Some examples of specific MIPS include: accurate self-perceptions (managing-self), communicating clear goals and expectations (communicating), building/maintaining relationships (supporting), considering employee differences when giving recognition/rewards (motivating), and detecting emerging conflicts (managing conflict). An interesting feature of interviewee responses across the higher-order themes was one of individualizing and tailoring one’s interpersonal interactions to specific employee backgrounds, traits, needs, values and beliefs.

The five higher-order themes identified in our qualitative study are consistent with the broader literature on workplace interpersonal skills and leader competencies. As above, some of the key themes identified in the literature included communication, relationship development, persuasion and influence, and conflict management. Communication skills seem essential to interpersonal effectiveness. Relationship development is highly overlapping with supportive behavior, and there is an extensive literature on the importance of social support in the workplace (e.g., Kossek et al., 2011). Persuasion and influence overlaps with motivating behaviors, and the latter may be broader in nature than, or may encapsulate, persuasion and influence, which harks back to our arguments regarding the importance of bandwidth fidelity (Hogan and Holland, 2003). Both the extant literature and our interviews identified conflict management as essential to managerial interpersonal skills. Consequently, our original item pool was informed by themes from the existing literature as well as our qualitative data.

Based on work to this point, our preliminary definition of MIPS is “*those skills that help managers understand, communicate with, motivate and influence others, and resolve conflicts in goal-directed organizational settings*.” Our preliminary conceptualization is that MIPS is a superordinate latent variable indicated by five factors, each indicated by specific items representing aspects of each skill set. A construct is multidimensional when it has multiple interrelated, yet separate, dimensions that represent one theoretical concept, which does not exist separately from its dimensions (see Edwards, 2001). A superordinate construct is indicated by its dimensions, i.e., the flow is from the construct to its dimensions, which is similar to a reflexive measure whereby items serve as indicators of the construct (Edwards, 2001). This is also the case with the Big Five model of personality in that each of the five factors indicates a higher-order latent factor (see Gosling et al., 2003). Our preliminary model posits that the superordinate construct *managerial interpersonal skills* is indicated by five separate yet interrelated dimensions, each of which is indicated by multiple skill-based items.

We sought to operationalize and assess this construct across four studies. First, we developed and refined a preliminary measure (Studies 1 and 2). We then assessed the construct and criterion validity of the measure (Study 3). Finally we conducted our main validation study with a multilevel matched supervisor-subordinate sample (Study 4). Our procedures across these four studies were designed to be consistent with other scale development papers published in top organizational psychology journals (e.g., Ferris et al., 2005; Yang et al., 2019). All four studies were IRB approved as minimal risk with participant consent obtained.

## Study 1

### Purpose, Sample, and Procedure

The goal of Study 1 was to test the statistical properties of our initial item pool, reduce that item pool based on the preliminary factor structure of the items, and conduct a preliminary investigation of the construct validity of our measure. Specifically, Study 1 was designed to probe (a) whether a 5 factor model seemed to fit the data best and (b) whether certain items seem to hang together more closely based on item content/wording, so we could use those items in further confirmatory studies.

Undergraduate and graduate business students at a large, comprehensive university in the Western United States completed an online survey, which asked them to answer questions about their current manager, as well as questions about their background and current job. The survey included all of our preliminary MIPS items. Only students with work experience were included in the survey. Participants were diverse: 56% female, 38% White, 27% Asian or Asian Americans, 23% Hispanic, 4.5% Black, and the rest indicated Middle Eastern or Other. They averaged about 1.9 years of work experience with their referent supervisor and had been in their jobs about 2.5 years. Participants responded to questions about their supervisor using a five point scale (1 = *not very true of my supervisor*; 5 = *very true of my supervisor*). The final sample included 312 respondents.

We followed a two-step approach to the evaluation of our preliminary items. In the first step, we used standard item analysis techniques to identify and remove any poorly performing items. For instance, we computed item-total correlations, alpha if item deleted, and standard deviations (Allen and Yen, 2002). We also scrutinized each item for face validity. In this step, we reduced the original pool of 100 items to 49 items. In the second step, we ran an exploratory factor analysis (EFA) (varimax rotation) on the retained items and three factors with eigenvalues greater than one emerged. The first factor (*supporting*) explained 36% of the variance, the second (*motivating*) explained 27% of the variance, and the third (*managing conflict*) explained 25%. Varimax rotation indicated there was high interfactor correlation and some cross-loading of items on these key factors, suggesting a potential over-arching factor. Our EFA employed a 6.24:1 ratio of sample to items, which according to Costello and Osborne (2005) analysis is likely to result in no more than one misclassified item on average and to lead to the correct factor structure around half of the time.

We had originally expected there to be five factors for our MIPS model. This exploratory study allowed us to draw three specific conclusions about the factor structure of our model. First, three factors emerged from the data, and these factors, after rotation, seemed to best fit *supporting*, *motivating* and *managing conflict*.

Second, the items representing *communication* did not load on a single factor; instead, these items cross-loaded on all three factors. Thus, we inferred that *communication* may be important to specific MIPS, but is not a distinct factor itself. In other words, *communication* is integral to how managers support, motivate, and manage conflict, though not as a separate factor. We believe that this is an important distinction, and a novel finding for the managerial skills literature, which we discuss in detail in later sections.

Third, we found that the items representing *managing-self* did not load consistently onto a single factor. We concluded that managing-self, although important to the development of interpersonal skills, is more fittingly abstracted as intrapersonal rather than interpersonal in nature. This result was more consistent with our literature review. As we explain in more detail in our discussion section, *managing-self* may be more appropriately conceptualized as an antecedent of MIPS as opposed to a separate dimension of MIPS (Whetten and Cameron, 2014). To examine this conclusion, we include *managing-self* as a possible predictor of our MIPS construct in Study 2.

We accordingly chose to move forward with a three-dimensional model and measure of MIPS (i.e., supporting, motivating and managing conflict). This involved retaining items with the highest loadings on these three factors, and removing items exclusively focused on managing-self and communicating (i.e., that lacked face validity for the remaining three MIPS dimensions). This left us with 6 items for *supporting*, and 7 each for *motivating* and *managing conflict*. Given our EFA results, we also slightly modified some items to ensure each dimension is indicated by communication (e.g., “communicates clear goals and expectations” for the *motivating* dimension). After reconceptualizing the preliminary three-factor multidimensional structure of our measure, we ran a second study to establish the construct validity of our refined measure.

## Study 2

The purpose of Study 2 was to test the three-dimensional factor structure of our refined item pool using confirmatory factor analysis (CFA) (narrowed to 20 items based on the results of Study 1), and to test the convergent and criterion-related validity of our preliminary measure. At this point in our item development and testing process, we had 20 items to assess MIPS: 6 for *supporting*, 7 for *motivating*, and 7 for *managing conflict*. Contemporary models posit that personality traits influence or are related to interpersonal skills (Morgeson et al., 2005). Thus, when examining the validity of a measure of MIPS, it is important to examine relationships between these skills and measures of personality. As mentioned above, we also use this opportunity to clarify the role of managing-self as an intrapersonal person-based antecedent to MIPS.

We also investigated whether MIPS predicted job satisfaction over and above relevant Big Five personality dimensions (i.e., agreeableness, extraversion) (McCrae and Costa, 1987). There is surprisingly little research connecting managers’ interpersonal skills to job attitudes in general, and no research to our knowledge connecting employee job attitudes to their perceptions of their manager’s interpersonal skills. Based on JD-R theory, traits can act as personal job resources that are buffers to strain (Bakker and Demerouti, 2017). It is important to establish the potential incremental validity of MIPS as a job resource beyond traits. Conversely, low levels of MIPS can be viewed as a job stressor – and job stressors predict job attitudes (Rudolph et al., 2016).

### Sample and Procedure

Graduate business students at a large, comprehensive university in the Western United States completed an online survey, which asked participants to answer questions about their current manager, their background and current job. The survey included our preliminary 20-item MIPS measure, measures of agreeableness and extraversion, as well as job satisfaction. Only students with work experience were included in the survey resulting in a final sample of 157 who completed the questionnaire. Participants were 62% female, 35% White, 32% Asian or Asian Americans, 27% Hispanic, 1.9% Black, and the rest indicated Middle Eastern or Other. They had worked about 2 years with their current supervisors and had been in their jobs about 3.5 years.

MacCallum et al. (1999) noted the congruence between reported and actual CFA results approaches 1:00 when the ratio between items and factors is 20:3 and the sample size is between 100 and 200 when communalities are high, which aligns with our hypothesized factor structure, our Study 2 sample size, and the factor loadings/communalities we observed in our Study 1 EFA.

### Measures

#### Managerial Interpersonal Skills

Managerial interpersonal skills (with supervisor as referent) were measured using the three-factor (refined) measure developed in Study 1. We had 6 items for supporting 7 each for motivating and managing conflict. See *Results* below for internal consistency reliability (i.e., Cronbach’s alpha) estimates. Example items for each factor, respectively, include: “Shows concern for employee well-being,” for *supporting*; “Encourages employees with specific ways they can improve their performance,” for *motivating*; and “Diffuses emotionally charged situations effectively” for *managing conflict*” (1 = *not very true of my manager*; 5 = *very true of my manager*).

#### Managing-Self

Managing-self (with supervisor as referent) was measured using an 8-item scale developed in study 1. An example item is “Takes responsibility for his or her own decisions” (α = 0.94). (1 = *not at all true of my manager*; 5 = *very true of my manager*).

#### Agreeableness of One’s Supervisor

Agreeableness of one’s supervisor was measured using four items from the mini-IPIP (Donnellan et al., 2006). Participants were asked to what degree each statement reflects their current supervisor, e.g., is “[My supervisor] sympathizes with other’s feelings” (1 = *very inaccurate*; 5 = *very accurate*) (α = 0.81).

#### Extraversion of One’s Supervisor

Extraversion of one’s supervisor was measured using four items from the mini-IPIP (Donnellan et al., 2006). An example item is “[My supervisor] is the life of the party” (1 = *very inaccurate*; 5 = *very accurate*) (α = 0.71).

#### Job Satisfaction of the Participant

Job satisfaction of the participant was measured using the 3-item scale from Judge et al. (1998). An example item is “I feel fairly well satisfied with my current job” (1 = *strongly disagree*; 5 = *strongly agree*) (α = 0.92).

#### Experience With One’s Supervisor

Participants were asked to indicate the total amount of time (in units of 3 month intervals) they had worked with their referent supervisor to control for variation in their knowledge of their supervisors’ personality and skills.

### Results

Table 1 displays bivariate correlations between MIPS and variables of interest.

**TABLE 1:** Means, standard deviations, reliabilities and correlations – Study 2.

Variables	M	SD	1	2	3	4	5	6	7	8
Supporting	3.75	0.98	*(0.92)*							
Motivating	3.42	1.02	0.82^∗∗∗^	*(0.92)*						
Managing Conflict	3.57	0.97	0.77^∗∗∗^	0.74^∗∗∗^	*(0.90)*					
MIPS	3.57	0.90	0.94^∗∗∗^	0.92^∗∗∗^	0.90^∗∗∗^	*(0.96)*				
Extraversion	3.51	0.82	0.40^∗∗∗^	0.39^∗∗∗^	0.27^∗∗∗^	0.39^∗∗∗^	*(0.71)*			
Agreeableness	3.60	0.81	0.65^∗∗∗^	0.54^∗∗∗^	0.55^∗∗∗^	0.63^∗∗∗^	0.56^∗∗∗^	*(0.81)*		
Job Satisfaction	3.69	0.95	0.59^∗∗∗^	0.56^∗∗∗^	0.55^∗∗∗^	0.61^∗∗∗^	0.24^∗∗^	0.37^∗∗∗^	*(0.92)*	
Managing-self	3.78	0.90	0.87^∗∗∗^	0.82^∗∗∗^	0.85^∗∗∗^	0.91^∗∗∗^	0.33^∗∗∗^	0.59^∗∗∗^	0.54^∗∗∗^	*(0.94)*
Experience with Supervisor	7.60	6.28	–0.08	–0.11	–0.03	–0.09	–0.05	–0.10	0.09	–0.07

*N = 157; ^∗∗^p < 0.01; ^∗∗∗^p < 0.001. Cronbach’s alpha’s in parentheses on the diagonal. All variables except Job Satisfaction and Experience with Supervisor were answered by subordinates with supervisor as referent. MIPS = Managerial Interpersonal Skills (Supporting, Motivating, Managing Conflict aggregated).*

#### Managerial Interpersonal Skills Reliability

The reliability for each of the three dimensions were substantially higher than the established rule of thumb of 0.70 (*supporting*, α = 0.92; *motivating*, α = 0.92; *managing conflict*, α = 0.90).

#### Managerial Interpersonal Skills Validity: Confirmatory Factor Analysis (CFA)

To confirm the multidimensional factor structure of MIPS, we ran a CFA. The hypothesized three-factor model, with a superordinate latent MIPS factor upon which the three sub-factors loaded, had acceptable fit indices (*X*^2^(167) = 367.88, CFI = 0.93, TLI = 0.92, SRMR = 0.048, RMSEA = 0.088) and significantly better fit (Δ*X*^2^ = 111.88, df = 3) than a one-factor model (*X*^2^(170) = 479.759, CFI = 0.89, TLI = 0.88, SRMR = 0.052, RMSEA = 0.11). All items for the hypothesized model loaded strongly onto their factors (loadings ranged from 0.70 to 0.91). Each dimension also loaded strongly on the superordinate MIPS factor (supporting = 0.94; motivating = 0.96, managing conflict = 0.95). Inter-correlations for each dimension were strong ranging from 0.74 to 0.82.

For validation purposes, our sample size prevented us from testing a model with a latent superordinate MIPS factor using a structural equation modeling approach for our subsequent analyses given our relatively small sample size (*n* = 157) and the numbers of parameters that would need to be estimated (although we do so in our main validation study, Study 4). Thus, given the high alphas for each subscale, strong inter-correlations between each dimension, and the results of our CFA, we created an aggregated measure. First, we aggregated each of three subscales as an average of the item scores for each factor, then we aggregated those subscales into an overarching, umbrella dimension and used it in our analyses of convergent and criterion-related validity. Table 1 displays reliability coefficients and correlations of Study 1 variables.

#### Convergent Validity

First, we examined correlations between the overall MIPS variable and the two dimensions of the Big Five. Results indicate that MIPS is correlated with both agreeableness (*r* = 0.63, *p* < 0.001) and extraversion (*r* = 0.39, *p* < 0.001). It also is highly correlated with managing-self (*r* = 0.91, *p* < 0.001). This is not surprising since these items initially were developed to be part of our MIPS measure; that said, we wanted to include this in our regression models in this study to test our proposition that managing-self may be a precursor or antecedent of MIPS, rather than a component skill, which we discuss in more detail later.

#### Criterion-Related Validity

To assess incremental criterion-related validity, we first regressed job satisfaction on agreeableness, extraversion, and managing-self, as well as experience with one’s supervisor. We ran a second step where we added the MIPS scale and calculated (a) whether MIPS was statistically significant and (b) whether the change in *R*^2^ was significant when MIPS was added to the regression model. Results indicate that MIPS is significantly related to job satisfaction (*B* = 0.73, *p* < 0.01) controlling for these other variables. MIPS also added significant incremental validity, at ρ*R*^2^ = 0.07 (*F* [1,155] = 20.63, *p* < 0.01). This result presented in Table 2 provides preliminary support for the criterion-related and incremental validity of our MIPS measure and supports a key proposition of JD-R theory, i.e., that job resources are important in addition to personal resources in predicting job attitudes (Bakker and Demerouti, 2017).

**TABLE 2:** Criterion-related validity – Study 2.

DV: Job Satisfaction	B	SE	95% CI	B	SE	95% CI
Constant	1.14^∗∗^	0.35	0.44, 1.83	1.24^∗∗^	0.34	0.58, 1.90
Experience with Supervisor	0.02	0.01	−0.00, 0.25	0.02^∗^	0.01	0.00, 0.04
Extraversion	0.06	0.10	−0.14, 0.25	0.02	0.09	−0.16, 0.20
Agreeableness	0.06	0.11	−0.16, 0.29	−0.03	0.10	−0.24, 0.18
Managing-self	0.52^∗∗^	0.10	0.33, 0.72	−0.07	0.17	−0.40, 0.27
MIPS				0.73^∗∗^	0.16	0.41, 1.04
*R*^2^	0.32^∗∗^			0.39^∗∗^		

N = 157; * p < 0.05, ** p < 0.01. All variables except Job Satisfaction and Experience with Supervisor were answered by subordinates with supervisor as referent. MIPS = Managerial Interpersonal Skills(Supporting, Motivating, Managing Conflict aggregated).

## Study 3

After establishing the preliminary factor structure of our items, we further tested the validity of our three-factor model with a separate student sample. Study 3 used the same item pool to assess MIPS as Study 2 with a few minor exceptions. First, we added one item for *supporting* in order to have 7 items for each scale. We also sought expert advice from eight leading scholars who have published research on MIPS or related constructs (e.g., emotional intelligence, political skills, social skills) to ensure that each item was worded as precisely and effectively as possible^6^.

The purpose of Study 3 was to further validate and refine our preliminary measure, and assess predictive and discriminant validity using a different sample, including measures of both employee and managerial performance. We again tested our over-arching proposition that MIPS function as a key job resource for employees. Undergraduate and graduate business students at a large, comprehensive university in the Western United States completed an online survey, which asked respondents to respond to questions about their current manager. The survey included our preliminary MIPS measure, measures of employee job attitudes, employee self-reported extra-role job performance, and employee perceptions of their manager’s job performance as criteria. We tested whether MIPS predict job attitudes and performance controlling for participants’ perceptions of their manager’s conflict avoidance, supervisor support, and leader-member exchange.

Previous research has shown that individual’s social skills predict job performance (e.g., Ferris et al., 2001). That said, prior research has not demonstrated that individual’s perceptions of their manager’s interpersonal skills are related to their own job performance or their perceptions of their manager’s performance. As was demonstrated in Study 2, MIPS are related to job attitudes, such as job satisfaction, above and beyond personal resources, namely personality traits. Since MIPS are predictive of job attitudes, they should also be predictive of job performance, which is consistent with the “motivational pathway” in JD-R theory (Bakker and Demerouti, 2014). Moreover, when employees feel understood and relationally connected to their supervisors (supporting), when they experience a match between their capabilities and work assignments (motivating), and when they believe their supervisors have the requisite skills to navigate workplace tensions (managing conflict), their basic psychological needs will be supported, and hence they should perform well (Schaufeli and Bakker, 2004). Since supervisor support (Kossek et al., 2011) and leadership are related conceptually to MIPS, and have been found to be important job resources for employees (e.g., Volmer et al., 2012), we deemed it essential to establish the incremental validity of MIPS over and above measures of these constructs.

Only student participants with work experience were included in the survey. The final sample of 124 participants (after listwise deletion) had an average of 8 years of experience, including about 2 years with their referent supervisor. About 57% of the sample were female; 57% were Asian (3), 29% = White, 11% Hispanic, and the rest identified as Middle Eastern or Black/African American. As in Study 2, our sample size and factor structure fits the expected statistical power needed to observe the “correct” factor structure of a model (MacCallum et al., 1999).

### Measures

#### Managerial Interpersonal Skills

Managerial interpersonal skills was measured using a 21 item scale refined in Study 2 and modified slightly for Study 3 based on additional expert input noted above (i.e., very minor wording changes and one additional item for supporting), resulting in 7 items each for supporting, motivating and managing conflict (1 = *not at all true of my supervisor*; 5 = *very true of my supervi*sor) (α = 0.90 to 0.92).

#### Conflict Avoidance

Conflict avoidance was measured using the scale developed by De Dreu et al. (2001) adapted to reflect the employee’s perspective of the supervisor. An example item is “My manager avoids confrontation about our differences” (1 = *strongly disagree*; 5 = *strongly agree*) (α = 0.87).

#### Supervisor Support

Supervisor support was measured using the three-item scale developed by Caplan et al. (1975). An example item is “My manager is willing to listen to my job-related problems” (1 = *to a very little extent*; 5 = *to a very great extent*) (α = 0.89).

#### Organizational Commitment

Organizational commitment of the participant was measured using the three-item affective commitment scale developed by Meyer et al. (1993). An example item is “I feel a strong sense of belonging to the organization” (1 = *strongly disagree*; 5 = *strongly agree*) (α = 0.89).

#### Supervisor Performance

Supervisor Performance of the supervisor was measured using the six-item task performance scale developed by Varma et al. (1996). An example item is “How would you rate his/her quality of work?” This measured employee’s perceptions of their manager’s job performance (1 = *very poor*, 2 = *marginal*, 3 = *average*, 4 = *good*, 5 = *excellent*) (α = 0.90).

#### Organizational Citizenship Behavior

Organizational citizenship behavior (OCB) measured extra-role performance of the participant using the seven-item OCB scale developed by Masterson et al. (2000). An example item is “I go out of my way to help coworkers with work-related problems” (1 = *strongly disagree*; 5 = *strongly agree*) (α = 0.86).

#### Leader-Member Exchange

Leader-Member Exchange (LMX) was measured using six items from the LMX-7 developed by Graen and Uhl-Bien (1995)^7^. An example item is “Does your supervisor usually know your level of satisfaction with your performance?” Another is “Do you believe your supervisor would defend and/or justify your decisions even when you were not present?” (1 = *rarely*, 2 = *ocassionally*, 3 = *sometimes*, 4 = *fairly often*, 5 = *very often*) (α = 0.91).

#### Experience With Supervisor

We again controlled for the total amount of time (in units of 3 month intervals) participants had worked with their referent supervisor.

## Results

Table 3 displays bivariate correlations for Study 3 variables.

**TABLE 3:** Means, standard deviations, reliabilities and correlations – Study 3.

Variable	M	SD	1	2	3	4	5	6	7	8	9	10
Supporting	3.59	0.93	*(0.91)*									
Motivating	3.16	0.96	0.78^∗∗∗^	*(0.91)*								
Managing Conflict	3.35	0.94	0.82^∗∗∗^	0.87^∗∗∗^	*(0.92)*							
MIPS	3.36	0.89	0.92^∗∗∗^	0.94^∗∗∗^	0.96^∗∗∗^	*(0.96)*						
Supervisor Support	3.57	1.02	0.86^∗∗∗^	0.75^∗∗∗^	0.79^∗∗∗^	0.85^∗∗∗^	*(0.89)*					
Conflict Avoidance	2.95	0.89	0.18^∗^	0.04	0.04	0.09	0.18^∗^	*0(.87)*				
OCB	3.95	0.66	0.35^∗∗∗^	0.26^∗∗^	0.29^∗∗∗^	0.32^∗∗∗^	0.29^∗∗∗^	0.25^∗∗^	*(0.86)*			
Supervisor Performance	3.71	0.84	0.68^∗∗∗^	0.71^∗∗∗^	0.75^∗∗∗^	0.75^∗∗∗^	0.68^∗∗∗^	0.12	0.22^∗^	*(0.90)*		
Organizational Commitment	3.27	0.97	0.39^∗∗∗^	0.37^∗∗∗^	0.33^∗∗∗^	0.38^∗∗∗^	0.40^∗∗∗^	0.13	0.30^∗∗∗^	0.33^∗∗∗^	*(0.89)*	
Leader-Member Exchange	3.21	0.99	0.77^∗∗∗^	0.72^∗∗∗^	0.73^∗∗∗^	0.78^∗∗∗^	0.76^∗∗∗^	0.20^∗^	0.27^∗∗^	0.69^∗∗∗^	0.42^∗∗∗^	*(0.91)*
Experience with Supervisor	8.09	6.89	0.02	–0.12	0.04	–0.03	0.04	–0.00	0.17	0.01	0.04	0.04

*N = 124; ^∗^p < 0.05; ^∗∗^p < 0.01; ^∗∗∗^p < 0.001. Cronbach’s alpha’s in parentheses on the diagonal. All variables except for OCB and Organizational Commitment were answered by subordinates with the supervisor as referent. MIPS = Managerial Interpersonal Skills (Supporting, Motivating, Managing Conflict aggregated); OCB = Organizational Citizenship Behavior.*

### Managerial Interpersonal Skills Reliability

Reliability for each of the three dimensions was above the established rule of thumb of 0.70 (*supporting*, α = 0.91; *motivating* α = 0.91; *managing conflict* α = 0.92).

### Managerial Interpersonal Skills Validity: Confirmatory Factor Analysis

To confirm the multidimensional factor structure of MIPS, we again ran a CFA. For a one-factor model (with all items loading on one single factor), fit indices were: (*X*^2^(189) = 540.89, CFI = 0.84, TLI = 0.83, SRMR = 0.061, RMSEA = 0.12). The hypothesized three-factor model, with a superordinate latent MIPS factor upon which the three sub-factors loaded, had fit indices (*X*^2^(186) = 468.55, CFI = 0.87, TLI = 0.86, SRMR = 0.056, RMSEA = 0.10) that significantly better fit the data than the one-factor model (Δ*X*^2^ = 72.34, df = 3). As Table 4 displays, all items loaded strongly onto their factors, and each factor loaded strongly on a superordinate MIPS factor. Similar to Study 2, inter-correlations for each dimension were strong ranging from 0.78 to 0.87.

**TABLE 4:** Study 3: Confirmatory Factor Analysis Loadings.

Item	Factor 1 (Supporting)	Factor 2 (Motivating)	Factor 3 (Managing Conflict)
S1	0.67		
S2	0.75		
S3	0.83		
S4	0.77		
S5	0.74		
S6	0.74		
S7	0.75		
M1		0.72	
M2		0.73	
M3		0.84	
M4		0.80	
M5		0.80	
M6		0.71	
M7		0.81	
MC1			0.83
MC2			0.81
MC3			0.82
MC4			0.73
MC5			0.74
MC6			0.81
MC7			0.81
MIPS Factor	0.90	0.95	1.00

*N = 124.*

Given our relatively small sample size (*n* = 138), we were unable to use a model with a latent superordinate MIPS factor using a structural equation modeling approach for our subsequent predictive analyses due to the number of parameters that would be estimated (although we do so in our main validation study, Study 4). Thus, given the high alphas for each subscale, high inter-correlations between each dimension, and the results of our CFA, we used an aggregated MIPS measure for our analyses of convergent and criterion-related validity. First, we aggregated each of three subscales as an average of the item scores for each factor, then we aggregated those subscales into an overarching, umbrella dimension.

### Criterion-Related Validity

Table 5 displays our criterion-related validity analysis results. For incremental criterion-related validity, we first regressed each of our dependent variables (organizational commitment, task performance and OCB) on experience with supervisors, conflict avoidance, LMX, and supervisor support. Next, we added the MIPS scale in a second step. Table 6 presents results of the final step. Results indicate that although MIPS was not significantly related to organizational commitment when added to the regression, it explained unique variance in perceptions of supervisor job performance (*B* = 0.55, *p* < 0.01; ρ *R*^2^ = 0.07, *F*[1,118] = 19.84, *p* < 0.01) and self-reported OCB (*B* = 0.27, *p* < 0.05; ρ *R*^2^ = 0.03, *F*[1,118] = 3.95, *p* < 0.05).

**TABLE 5:** Criterion-Related Validity with MIPS – Study 3.

	Organizational Commitment				Supervisor Performance				OCB			
	B	SE	95% CI		B	SE	95% CI		B	SE	95% CI	
			Lower	Upper			Lower	Upper			Lower	Upper
Constant	1.60**	0.40	0.81	2.39	1.17**	0.25	0.67	1.67	2.61**	0.28	2.07	3.16
Experience w/Supv.	0.00	0.01	−0.02	0.03	0.00	0.01	−0.01	0.02	0.02	0.01	0.00	0.03
Conflict Avoidance	0.05	0.09	−0.13	0.24	0.02	0.06	−0.10	0.14	0.18**	0.06	0.05	0.30
Supervisor Support	0.15	0.16	−0.16	0.48	−0.02	0.10	−0.22	0.18	−0.04	0.11	−0.26	0.18
LMX	0.24	0.14	−0.03	0.51	0.21*	0.08	0.04	0.38	−0.03	0.09	−0.22	0.15
**MIPS**	**0.04**	**0.20**	**−0.35**	**0.43**	0.55**	**0.12**	**0.31**	**0.80**	0.27*	**0.14**	**0.00**	**0.54**
*R*^2^	0.19				0.58				0.17			

N = 124 (after listwise deletion); *p < 0.05, **p < 0.01; LMX = Leader-Member Exchange; MIPS = Managerial Interpersonal Skills (Supporting, Motivating, Managing Conflict aggregated).

**TABLE 6:** Study 4: CFAs with Level 1 Employee Ratings and Level 2 Supervisor Self Ratings of MIPS.

Employee Level				Supervisor Level			
Item	Factor 1 (Supporting)	Factor 2 (Motivating)	Factor 3 (Managing Conflict)	Item	Factor 1 (Supporting)	Factor 2 (Motivating)	Factor 3 (Managing Conflict)
S1	0.91			S_S1	0.61		
S2	0.92			S_S2	0.62		
S3	0.90			S_S3	0.49		
S4	0.92			S_S4	0.76		
S5	0.90			S_S5	0.63		
S6	0.90			S_S6	0.75		
S7	0.89			S_S7	0.61		
M1		0.89		S_M1		0.50	
M2		0.92		S_M2		0.77	
M3		0.91		S_M3		0.66	
M4		0.90		S_M4		0.46	
M5		0.92		S_M5		0.64	
M6		0.85		S_M6		0.64	
M7		0.89		S_M7		0.76	
MC1			0.92	S_MC1			0.77
MC2			0.91	S_MC2			0.73
MC3			0.93	S_MC3			0.83
MC4			0.86	S_MC4			0.65
MC5			0.92	S_MC5			0.68
MC6			0.92	S_MC6			0.63
MC7			0.94	S_MC7			0.82
	**Superordinate MIPS factor loadings**				**Superordinate MIPS factor loadings**		
S	0.96			S	0.78		
M	0.96			M	0.88		
MC	0.94			MC	0.96		

*For Employee-level, N = 566, For Supervisor-level, N = 144; ^∗^p < 0.05, ^∗∗^p < 0.01; MIPS = Managerial Interpersonal Skills.*

### Studies 1–3 Discussion

Based on our review of the literature on workplace and managerial interpersonal skills, and semi-structured interviews with practicing managers, we initially proposed a multidimensional model of MIPS with five factors, managing-self, communicating, supporting, motivating, and managing conflict, each indicated by various items representing aspects of each skill. Items from each of the five dimensions demonstrated high levels of internal consistency reliability. Contrary to our expectations, we found a five-factor solution was not the best fit to the data. In fact, a three-factor model was the best fit to the data with supporting, motivating and managing conflict as distinct factors. Communication skills are an important aspect of each of these three dimensions, along with individualizing one’s interpersonal exchanges with subordinates across all three dimensions—what we call *individuation*. Furthermore, managing self did not appear to represent a distinct factor and was more appropriately conceptualized as an intrapersonal versus an interpersonal skill. These results yielded a slightly modified working definition of MIPS to include *skills that help managers support and motivate others, and constructively resolve conflicts in goal-directed organizational setting*s. Implicit in this definition is that effective communication and active listening skills, along with individuation, are integral to MIPS.

Studies 1, 2, and 3 provide preliminary evidence for a model of MIPS with three dimensions (supporting, motivating, managing conflict). Each of these three factors is indicated by items that include communication, and individuation, i.e., individualizing interpersonal exchanges with others. Results also suggest that our MIPS measure is significantly related to job attitudes (i.e., job satisfaction, Study 2) over and above personality traits, and to perceptions of supervisor job performance, and self-reported OCBs—over and above leadership perceptions (LMX), employee perceptions of supervisor support and conflict avoidance (Study 3).

In both studies 2 and 3, we control for the number of years an employee has been supervised by his/her supervisor to account for how much knowledge of their supervisor’s skills they may or may not have. We selected other variables based on JD-R theory—personality traits that may influence how a person rates their supervisor (agreeableness, extraversion; Study 2), other evaluations of relationships with one’s supervisor (LMX, Supervisor Support, Supervisor Conflict Avoidance; Study 3)—to evaluate whether MIPS is associated with key employee outcomes over and above other important personal and job resources. At this point, we still consider our measure as preliminary. Although the MIPS three factor and superordinate factor structure fit the data well, we treated the measure as unidimensional in studies 2 and 3 due to relatively smaller sample sizes. To be more confident in the factor structure and criterion-related validity of our MIPS Scale, we collected additional data with a larger sample of full time employees and their managers and tested the full factor structure with a multilevel SEM model.

## Phase 3: Validation of the Managerial Interpersonal Skills (MIPS) Scale-Study 4

Consistent with studies published in leading organizational psychology journals (e.g., Ferris et al., 2005; Yang et al., 2019), the purpose of Study 4 was to build on our prior results to further validate our MIPS Scale. Results from studies 1 to 3 suggest that MIPS has three dimensions: supporting, motivating and managing conflict, and a superordinate MIPS factor. In Study 4, we collected matched employee and supervisor data from a large healthcare organization in the Western United States^8^ to build on our prior studies in several key ways. First, our data are from working professionals embedded in the same organization. In this regard, we add the manager’s perspective to the prior employee perspective. Our intent was to extend our MIPS measure so that it can be used for both self-report (managers reporting on self as referent) and other-report (subordinates reporting on manager as referent) purposes. Second, we collected supervisor-subordinate matched data, which allows us to examine how MIPS are related to outcomes at multiple levels of analysis and mitigates potential concerns about same-source variance.

Specifically, from the employee perspective (Level 1) we examine how employee ratings of their managers’ MIPS predicts employees’ own organizational citizenship behaviors, while nested within a particular supervisory unit. We also examine whether a manager’s self-reported MIPS (Level 2) is related to their own job performance (as measured from the employees’ perspective). Given our findings in Studies 1 to 3, we chose our control variables carefully to further demonstrate the incremental validity of MIPS in predicting outcomes relevant to JD-R theory. At level 1, we control for employee perceptions of their supervisor’s social astuteness, interpersonal influence, and interpersonal justice. Social astuteness and interpersonal influence are “political skills” (Ferris et al., 2005) that could be considered important personal resources that are closely related to MIPS. Interpersonal justice is part of a social exchange evaluation of how fairly one’s manager treats them, building on study 2 where we assessed the effects of MIPS over and above LMX. With all three variables, we ask employees to rate their supervisor. At level 2, we control for the manager’s emotional intelligence and cultural intelligence to differentiate MIPS from alternative managerial abilities or personal resources.

Using multilevel modeling allows us to account for shared variance of employees nested in supervisory units. We first test a hierarchical nested confirmatory factor analysis for our MIPS measure to establish the validity of this measure in a nested research design. We then assess the criterion-related validity of our MIPS measure.

### Sample and Procedure

Surveys were completed by managers of each department of a large healthcare organization in Southern California. Supervisor surveys included a self-report measure of MIPS, among other measures, namely self-reports of emotional intelligence and cultural intelligence. Employees within each department also were sent surveys and asked to identify their supervisor (for purposes of matching data), and asked questions about their manager’s interpersonal skills, interpersonal justice, as well as their own job performance and their manager’s job performance.

We ran CFAs with larger samples of employees and supervisors to again validate the factor structure of our MIPS measure, and predictive models with smaller matched employee-supervisor samples to assess the convergent and criterion validity of MIPS. The larger sample for our CFAs without outcome variables included Level 1 employees (*N* = 566) and Level 2 supervisors (*N* = 144) (similar average cluster size as our structural equation models reported below). Our sample size for our CFAs again reaches expected levels of statistical power (> 0.80) based on MacCallum et al. (1999) for Level 1, and is similarly powerful for detecting the appropriate factor structure at Level 2.

For our models with outcome variables, we had a smaller final employee sample after listwise deletion and pairing with supervisors (*N* = 248). This sample had about 21 years of experience, including about 6.5 years with their current supervisor, and about 81% were female. About 25% of the sample were Asian, 43% identified as White, 26% as Hispanic, and the rest as either Middle Eastern or Black/African American. Among the final set of supervisors after listwise deletion (*N* = 66), 76% identified as female with about 3.76 matched employees reporting to each manager.

For our structural equation modeling, we referred to Scherbaum and Ferreter (2009) simulation regarding sample sizes required for multilevel analyses. For our final model, our supervisor level (Level 2) sample size (*N* = 66), and our Level 1 sample size is about *N* = 4 for each supervisor. This results in an approximate power between 0.75 and 0.80 for detecting a medium effect size. However, we also include covariates at each level, which increases power in multilevel analyses (Raudenbush, 1997). Thus, we are confident in our statistical power for our Level 1 and Level 2 CFAs, for our Level 1 outcomes, and we expect adequate power with Meuleman and Billiet (2009) recommended Level 2 threshold (*N* > 60) to detect large structural effects at the between level, though not for medium effects.

### Measures – Employees

#### Interpersonal Justice (About Manager)

Interpersonal justice (about manager) was measured using the four-item scale developed by Colquitt (2001). An example item is “To what extent has your manager treated you in a polite manner?” (1 = *to a very little extent*; 5 = *to a very great extent*).

#### Job Performance (About Manager)

Job performance (about manager) was measured using three items from the task performance scale developed by Varma et al. (1996), adapted to reflect the department manager’s performance. An example item is “How would you rate your department manager’s timeliness at work - in terms of completing assignments on time?” (1 = *poor*, 2 = *marginal*, 3 = *average*, 4 = *good*, 5 = *excellent*). These items were aggregated among the subordinates into an average rating for each manager (ICC by manager of 0.81), as this measure was used as a Level 2 (manager) level outcome.

#### Managerial Interpersonal Skills (About Manager)

Managerial Interpersonal Skills (about manager) – was measured using the same 21 items (7 each for *supporting*, *motivating* and *managing conflict*) used in Study 3. Employees rated their perceptions of their manager’s MIPS (1 = *not at all true of my manager*; 5 = *very true of my manager*).

#### Political Skill – Social Astuteness (About Manager)

Political skill – Social astuteness (about manager) was measured using the five-item scale developed by Ferris et al. (2005) adapted to reflect the employee’s perception about their manager. An example item is “My manager always seems to instinctively know the right thing to say or do to influence others” (1 = *strongly disagree*; 5 = *strongly agree*).

#### Political Skill – Interpersonal Influence (About Manager)

Political skill – Interpersonal influence (about manager) was measured using the four-item scale the scale developed by Ferris et al. (2005) adapted to reflect the employee’s perception about their manager. An example item is “My manager is able to communicate easily and effectively with others” (1 = *strongly disagree*; 5 = *strongly agree*).

#### Organizational Citizenship Behavior (About Employee-Self)

Organizational citizenship behavior (OCB) (about employee-self) was again measured using the scale developed by Masterson et al. (2000). An example item is “I go out of my way to help coworkers with work-related problems” (1 = *strongly disagree*; 5 = *strongly agree*).

### Measures – Managers

#### Cultural Intelligence (Metacognitive) (About Manager-Self)

Cultural intelligence (metacognitive) (about manager-self) was measured using three items from the subscale developed by Ang et al. (2007). An example item is “I change my non-verbal behavior when a cross-cultural situation requires it” (1 = *strongly disagree*; 5 = *strongly agree*).

#### Emotional Intelligence (Self-Control) (About Manager-Self)

Emotional intelligence (self-control) (about manager-self) was measured using the six-item TEIQ self-control scale developed by Cooper and Petrides (2010). An example item is “I’m usually able to find ways to control my emotions when I want to” (1 = *strongly disagree*; 5 = *strongly agree*).

#### Managerial Interpersonal Skills (About Manager-Self)

Managerial Interpersonal Skills (about manager-self) – was measured using the same items as those in Study 3, adapted for self-report (1 = *not at all true of me*; 5 = *very true of me*).

### Results

We do not provide a correlation matrix or internal consistency reliability estimates for Study 4 because all analyses were run using latent variables modeled by observed items^9^.

#### Managerial Interpersonal Skills Validity: Confirmatory Factor Analysis

To test the factor structure of the MIPS measure, we ran CFAs using four separate approaches for a comprehensive analysis. For all models, consistent with our prior studies, MIPS was conceptualized as items loaded on sub-scales, which were then loaded onto a superordinate MIPS factor.

(1) We modeled employee ratings of manager MIPS at both level 1 and 2 to capture the within- and between-portions of the measure. In multilevel language, this means we modeled the proportion of the variance in each item that existed at the individual level, and then modeled the random intercepts at the manager-level (if there is a systematic portion of it to model).
(2) We ran one-level CFAs with only manager self-ratings to model the structure of managers’ self-ratings only.
(3) We ran CFAs with managers’ self-ratings at the group-level and employee ratings of the manager at the individual level, which most accurately reflects our modeling of the MIPS construct—as reflecting an employee’s perception of a manager, which can vary across individual raters, and as reflecting a managers’ self-ratings of their own MIPS.
(4) We combined all of these approaches and modeled the within and between portions of the employee ratings *along with* managers’ self-ratings at Level 2.

#### Employee Ratings of MIPS on Levels 1 and 2

First, for employee ratings only, modeled at the within-manager and the between-manager levels, all items loaded onto their selected factors and those factors onto a superordinate MIPS factor, with adequate fit (CFI = 0.96, TLI = 0.96, RMSEA = 0.048, SRMR (within) = 0.047, SRMR (between) = 0.625). Between level factors represent the factor structure of the remaining random intercepts and can help us to understand whether there is a systematic shared factor structure of the means of the intercepts across managers. Our model fit at that level was relatively poor, at least in terms of the separate SRMR results, suggesting that the factor structure is, at the least, different at level 2 than at level 1 (i.e., the part of the MIPS construct that varies between managers is not the same as the part that varies within managers). Indeed, examination of factor loadings and modification indices suggest two specific items do not load well at the between-manager level: MC2 (‘‘Manages difficult situations with composure’’) and MC4 (‘‘Controls his or her emotions when managing conflict’’). Both were negatively loaded on the between-level MC factor, which is the source of the poor fit. Removing these items from the between-level MC factor improved the between-level SRMR [SRMR(between) = 0.158] and all items loaded strongly on their associated factors. This provides support for our overall factor structure using multilevel, clustered data.^10^

#### Manager Self-Ratings of MIPS on Level 2

Next, we ran the CFAs with only the manager self-ratings, at a single-level, with a superordinate MIPS factor. Given the relatively small sample size (*N* = 66), model fit was adequate (CFI = 0.88, TLI = 0.86, RMSEA = 0.079, SRMR = 0.081).

#### Employee Ratings of MIPS on Level 1 and Manager Self-Ratings on Level 2

**FIGURE 1:**
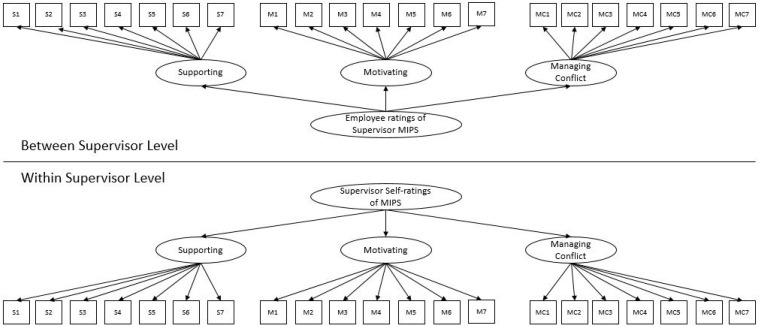
Study 4 multi-level measurement model.

Third, we included manager’s self-ratings and employee ratings of the manager, with employee ratings at level 1 only, and self-ratings at level 2, and with all items loaded onto their selected factors and those sub-factors onto a superordinate MIPS factor, the fit statistics indicated a good fit (CFI = 0.96, TLI = 0.95, RMSEA = 0.049, SRMR (within) = 0.021, SRMR (between) = 0.080). This is the most useful and rigorous model as it combines manager’s own self ratings with subordinate ratings of managers. Thus, this model accounts for variance between managerial clusters. Factor loadings for this model—which we used in structural equation models—are displayed in Table 6. Figure 1 also displays the measurement model.

#### Employee Ratings of MIPS on Levels 1 and 2 and Manager Self Ratings of MIPS on Level 2

Finally, we combined these three approaches and included the between-level factor of the employee ratings of MIPS along with the manager self-ratings of MIPS, and found good fit: CFI = 0.95, TLI = 0.95, RMSEA = 0.042, SRMR (within) = 0.022, SRMR (between) = 0.162. The between-portion of the employee ratings of MIPS (modeled as a superordinate factor) and the manager self-ratings of MIPS (modeled as a superordinate factor) were not correlated, suggesting that these two assessments captured something different depending on the rater. Combined, these four CFAs provide further support for our proposed factor structure, even at multiple levels of analysis and for both self- and other-ratings. In the multilevel structural equation modeling analyses that follow to assess convergent and criterion validity, we continue with the third measurement model as specified above and displayed in Table 6 and Figure 1.

### Multilevel Modeling

We modeled each MIPS item on its selected factor, and all items onto a second-order superordinate MIPS factor. With employee ratings at level 1 and managerial self-ratings at level 2, we tested the model above in a series of steps. Adding covariates at level 1, the model had an RMSEA of 0.043, CFI of 0.96 and TLI of 0.96, with an SRMR (within) of 0.026, and between of 0.084. MIPS was associated, via covariances between latent factors, with social astuteness (1.04, *p* = 0.00), interpersonal influence (1.084, *p* = 0.00), and interpersonal justice (0.80, *p* = 0.00), supporting convergent validity of our MIPS measure.

When we added our level 1 outcome variable to examine criterion validity, model fit was good (RMSEA = 0.04, CFI = 0.95, TLI = 0.95, SRMR (within) = 0.031, SRMR (between) = 0.08). Employee ratings of their managers’ MIPS predicted employees’ own OCBs (0.19, *p* = 0.00) beyond social astuteness (0.07, *p* = 0.65), interpersonal influence (−0.10, *p* = 0.20) and interpersonal justice (−0.02, *p* = 0.75), each of which were not related to OCBs when considering MIPS.

Next, we added managerial level 2 covariates and predictive validity of managerial self-ratings of MIPS (RMSEA = 0.06, CFI = 0.90, TLI = 0.89, SRMR (within) = 0.066, (between) = 0.173) showing manager self-ratings of MIPS predict employee-rated manager performance (0.68, *p* = 0.046) beyond emotional intelligence (0.04, *p* = 0.94) and cultural intelligence (−0.16, *p* = 0.19), controlling for manager self-reported experience (0.00, *p* = 0.03). Manager self-ratings of MIPS did not predict employee OCBs (−0.04, *p* = 0.91).

### Study 4 Discussion

This study provided important further validation of our MIPS measure. Our CFA again supported a three-factor model of MIPS with a superordinate factor. That said, when examining the factor structure of MIPS with both employee- and manager-reported data included simultaneously, the fit of the Level 2 (manager-level) model was not as good. Removal of two negatively loaded items improved fit substantially. Nonetheless, this provides ample opportunity to propose further ways in which the between-level composition of variance in employee ratings of MIPS might be understood. Since the between-manager factor structure in multilevel CFA reflects the common covariance in ratings of MIPS across raters, this can be modeled in the same way (e.g., a superordinate MIPS factor, with three sub-factors), or possibly in other ways (e.g., as just “MIPS” without sub-factors, as just sub-factors without a superordinate factor, etc.). None of this would invalidate our findings (which show adequate fit), but may indicate that future researchers should be sure to check the factor structure across levels when using this scale as a multi-level instrument.

Our results indicate employee reports of MIPS are related to, though distinct from, similar constructs, such as interpersonal influence and social astuteness, which supports the convergent and discriminant validity of our measure. Results also suggest that MIPS are related to OCBs, controlling for variables that would be expected to be correlated with MIPS. Furthermore, we found that manager’s self-reports of MIPS are related to their job performance as rated by their employees, controlling for related factors. This supports the criterion-related validity of MIPS, both as reported by employees and managers. These results also provide preliminary evidence that MIPS is related to important outcomes even when controlling for similar variables. Results also suggest that managerial self-ratings and employee ratings of MIPS have empirically distinct factor structures that are related to different outcomes. For a more comprehensive understanding of MIPS, it may be possible to use them together in future research.

## Overall Discussion

When we think of bad managers, we usually think of the so-called “jerks” (Casciaro and Lobo, 2005) who mistreat their employees interpersonally and contribute to a toxic work environment (Sutton, 2007). This depletes the resources that managers and their employees need for coping with the demands of their jobs. Our conceptualization of MIPS was grounded in JD-R theory (Bakker and Demerouti, 2014) because we believed that interpersonal skills are a key resource that help managers perform well. We also proposed that MIPS are a key resource whereby employees gain control over their work environment through the esteem, feedback and motivation necessary for them to perform well. As we discuss below, our findings generally supported these propositions. To our knowledge, ours is the first study to develop a model and validate a concomitant measure of MIPS. In so doing, we demonstrated evidence for the reliability, construct validity, criterion-related, and incremental validity of the MIPS Scale.

### Implications for Theory and Research

#### A Multidimensional Model of MIPS

One of our key contributions is to offer a conceptual model, definition, and measure of MIPS that is grounded in previous research, informed by practicing managers, and validated across several different samples from both employee and manager perspectives. We defined MIPS as *skills that help managers support and motivate others, and effectively resolve conflicts in goal-directed organizational settings.* Our results indicate that the MIPS construct is multidimensional, with three dimensions of supporting, motivating, and managing conflict indicating a superordinate latent MIPS factor, at least when measured in terms of the employees’ evaluation of their managers. From the manager’s perspective (i.e., self-report), evidence from one organization suggests that a three-factor model is the best fit to the data. The dimensionality of MIPS is similar to that of personality in that it is a latent factor indicated by several distinct subfactors, each important in its own right.

#### Managing-Self and Communicating

Our first pilot study revealed that items intended to indicate managing-self did not fit the data. In hindsight, this makes sense: self-management is needed to develop interpersonal skills, but is intrapersonal – not interpersonal – in nature (Whetten and Cameron, 2014). As such it is more appropriate to consider it as an antecedent to interpersonal skills. This diverges somewhat from the findings of our qualitative interviews with practicing managers, who consistently indicated managing-self is an important aspect of MIPS. It also highlights the importance of approaching our construct and measure development from both inductive and deductive approaches. Future research should more thoroughly examine the role of self-management in the development of interpersonal skills.

In Study 1, items intended to indicate communication also loaded on the other three factors (supporting, motivating, managing conflict), rather than emerging as a distinct factor. Initially this seemed inconsistent with prior research emphasizing communication as an important component of interpersonal skills in organizations (e.g., Klein et al., 2006). Qualitatively, our interviews with practicing managers similarly revealed communication was the skill most talked about. Quantitatively, our factor analytic results revealed communication as a key skill enabling managers to effectively support and motivate others and to manage conflict. Communication is apparently not a *separate* dimension of MIPS, but an important foundational element of other substantive managerial interpersonal skills. Thus, our final measures include items to assess interpersonal communication in each of the three dimensions of MIPS.

#### A Prospective Developmental Model of MIPS

Our results suggest an intriguing developmental model of MIPS, which if supported in future research has important implications for management and organizations (e.g., selection, training and development). That is, managers must first be self-aware and able to self-manage in order to be interpersonally skilled. Next, managers need to develop effective communication skills—both messaging and listening—as a foundational element of the three dimensions identified by our measure (supporting, motivating, and managing conflict). Furthermore, each skill dimension provides a critical, additive precursor. For instance, developing supportive relationships and demonstrating genuine concern for the well-being of subordinates is a necessary prelude to effectively motivating them. We suspect that if employees do not feel their manager is supportive, efforts to motivate those employees will be less effective. Finally, skillful motivation of others, such as tailoring feedback to individuals based on their unique backgrounds and understanding what makes others “tick,” is foundational for effective conflict management. Managing conflict involves understanding the distinctive interests, issues, and personalities of the individuals involved. We propose that managers who are more skillful at supporting and motivating will be better equipped to understand and manage individuals with conflicting personalities or concerns. Underlying each of these three dimensions is individuation, the acumen to individually tailor one’s interpersonal exchanges aimed at supporting, motivating and managing conflict.

#### Convergent Validity

Prior research has suggested that interpersonal skills are related to or influenced by personality traits (e.g., Morgeson et al., 2005; Klein et al., 2006). We found in Study 2 that personality traits of agreeableness and extraversion, perhaps the two traits of the Big Five that are most closely tied to interpersonal skills, were strongly correlated with managerial interpersonal skills. This helps to demonstrate the convergent validity of the MIPS scale. Given our results are based on only one study and two traits, the extent to which personality traits influence managerial interpersonal skills is an area for future research.

Our results suggest that managerial interpersonal skills are correlated with similar constructs. We expected that managers who are more interpersonally skilled should be more politically skilled and would be viewed as more interpersonally fair. In Study 4—our main validation study in a large healthcare organization—employee ratings of manager’s MIPS were related to, yet distinct from, manager’s political skills, namely social astuteness and interpersonal influence (Ferris et al., 2005), as well as interpersonal justice (see Colquitt, 2001). This provides additional evidence for the convergent, discriminant, and construct validity of our MIPS measure. Future research on the convergent validity of this new MIPS Scale, as well as its nomological network, would be useful.

#### Criterion-Related and Incremental Validity

Based on job demands-resources theory (Bakker and Demerouti, 2014), we proposed that managerial interpersonal skills function as a key job resource for employees (Perrewé et al., 2000, 2005), and that they should be positively related to employees’ job attitudes and performance (Demerouti et al., 2001). Our results largely support the JD-R model and our overarching proposition. In Study 2, employee ratings of their managers’ MIPS explained unique variance in job satisfaction beyond personality, an important personal resource, and experience with supervisors. In Study 3, employee reports of their managers’ MIPS explained unique variance in their own OCBs and ratings of supervisor performance, beyond supervisor social support and conflict avoidance. To our knowledge, ours is the first study to show that employee perceptions of MIPS are related to their own performance and perceptions of their manager’s performance. In Study 4, employee ratings of their manager’s MIPS predicted manager’s performance, controlling for manager’s self-reports of emotional and cultural intelligence. Overall, our results are consistent with the concept of bandwidth fidelity (Hogan and Holland, 2003; Kossek et al., 2011) in that MIPS were predictive of broad outcomes, such as job attitudes and performance, above and beyond constructs with less breadth in their scope, such as supervisor support.

#### Practical Implications

We hope that by developing and validating a measure of MIPS, organizations can use our measure for a variety of purposes such as identifying manager’s training and development needs, assessing MIPS training effectiveness, and potentially for selection into managerial positions once more research using the MIPS Scale has accumulated. In terms of training, previous research on MIPS has tended to focus either on very specific skills, such as assertive communication or broad leadership competencies that are not strictly interpersonal in nature (e.g., structuring, which involves prioritizing for instance). This is consistent with the broader applied psychology literature on interpersonal skills. Our model refocuses efforts on a narrower set of skills that have implications for managerial training. Namely, we suggest training should focus mainly on three component skills: supporting, motivating and managing conflict, with interpersonal communication skills and individuation integrated into each of the three components. For instance, training could focus on how to build supportive relationships through active listening, how to motivate employees by communicating clear goals and expectations, and how to manage conflict by effectively diagnosing sources of conflict and articulating solutions through effective problem solving. Each of these skills should be developed with attention and effort focused on the importance of an employee’s traits, preferences and values in how one relates, motivates, and manages conflicts. For instance, an employee who is more intrinsically motivated may require a different pattern of interactions than one who is more extrinsically motivated. Trainers also may consider focusing on developing self-awareness skills as a prerequisite to building effective MIPS.

There is limited research on interpersonal skills assessment for selection purposes. Lievens and Sackett (2012) found that a situational judgment test of medical school applicants’ procedural knowledge of interpersonal skills predicts internship performance and subsequent on-the-job performance up to nine years later (Lievens and Sackett, 2012). Research has also shown that although an overwhelming majority of graduate business programs consider applicant’s interpersonal skills as important, a small minority assess them (Beenen et al., 2018). There is relatively little research on the assessment of interpersonal skills for purposes of selection or placement into managerial positions specifically. Of course, to be used for selection purposes in any organizational setting, a selection tool must meet a number of rigorous standards, which are outlined in the Uniform Guidelines on Selection Procedures^11^. More research is needed using our MIPS measure before relying on it for selection purposes.

### Conclusion

Ours is the first measure developed to assess interpersonal skills that are specific to the managerial role. It is also the first measure of managerial or leadership interpersonal skills developed based on both inductive and deductive approaches (i.e., by qualitative inquiry with practicing managers and surveys from employees and their managers). Our goal was to develop a model of MIPS that was both comprehensive and specific and that mitigates the “challenging and sometimes frustrating” state of research on interpersonal skills (Spitzberg and Cupach, 2011, p.488). Given that our measure is the only one specifically designed to assess MIPS, and its development was informed by both prior research and by the experience of managers and executives, we hope it proves useful for a variety of practical purposes for managers and organizations.

## Data Availability Statement

The raw data supporting the conclusions of this article will be made available by the authors, without undue reservation.

## Ethics Statement

The studies involving human participants were reviewed and approved by California State University, Fullerton Institutional Review Board. The patients/participants provided their written informed consent to participate in this study.

## Author Contributions

GB: project conceptualization, co-principal investigator (Co-PI) on GMAC grant, data collection, preliminary data analysis, and co-lead on writing for main article. SP: project conceptualization, principal Investigator (PI) on GMAC grant, data collection, preliminary data analysis, and co-lead on writing for main article. BL: lead on database creations and data analysis, wrote results sections, created tables for results, and assisted in writing manuscript. RR: project conceptualization, advisor on grant, and assisted in writing manuscript. All authors contributed to the article and approved the submitted version.

## Conflict of Interest

The authors declare that the research was conducted in the absence of any commercial or financial relationships that could be construed as a potential conflict of interest.

## Footnotes

1 We would like to thank Kim Cameron, Cary Cherniss, Shane Connelly, Sean Hannah, Paulo Lopes, Bob Rubin, Robert Tett, and Darren Treadway for their time and helpful comments on our items.

2 This is across both blue- and white-collar work.

3 In our empirical research, we control for general supervisor support so as to demonstrate incremental related validity of our MIPS measure.

4 Readers interested in O^∗^Net can visit: https://www.onetonline.org.

5 Table 1, pg. 211.

6 In a few cases, we made minor changes to our items before moving forward with Study 3. For instance, for supporting, we refined one item from “is viewed as approachable by others” to “is approachable by others”. These reflected minor verbiage changes and not substantive changes to the content of the items themselves.

7 We did not use the global evaluation item (the 7th item).

8 The MIPS items used in Study 3 are the same as those in Study 2, with some minor changes, i.e., we added one item to the supporting and managing conflict factors.

9 A full item correlation matrix is available from the first author upon request.

10 Because, we did not include manager-level responses which reduced our sample due to matching, our sample size was markedly higher (*N* = 566, rating 144 managers as opposed to *N* = 248 matched with *N* = 66 managers). Listwise deletion makes the next analyses less comparable, as the sample sizes shift based on the manager response rate.

11 https://www.eeoc.gov/laws/regulations/
